# Biofilms and multidrug resistance: an emerging crisis and the need for multidisciplinary interventions

**DOI:** 10.3389/fbioe.2025.1625356

**Published:** 2025-06-25

**Authors:** Mohd Fakharul Zaman Raja Yahya, Mohd Taufiq Mat Jalil, Norashirene Mohamad Jamil, Nurul Hidayah Mohamad Nor, Nasser Alhajj, Rikson Siburian, Nazia Abdul Majid

**Affiliations:** ^1^ Faculty of Applied Sciences, Universiti Teknologi MARA Shah Alam, Shah Alam, Selangor, Malaysia; ^2^ Integrative Pharmacogenomics Institute (iPROMISE), Puncak Alam, Selangor, Malaysia; ^3^ Low Dimensional Materials Research Centre, Faculty of Science, Universiti Malaya, Kuala Lumpur, Malaysia; ^4^ Pharmaceutical and Molecular Biotechnology Research Centre (PMBRC), South East Technological University (SETU), Waterford, Ireland; ^5^ Faculty of Mathematics and Natural Sciences, Universitas Sumatera Utara, Medan, Indonesia; ^6^ Institute of Biological Sciences, Faculty of Science, Universiti Malaya, Kuala Lumpur, Malaysia

**Keywords:** biofilms, multidrug resistance, antibiotics, antibiofilm activity, natural antimicrobials, synthetic antimicrobials, nanoparticles, probiotics

## Abstract

The escalating prevalence of multidrug resistance (MDR) represents not merely a medical challenge, but a systemic shortcoming in our current antimicrobial paradigms. Central to this crisis are biofilms, the structured microbial communities that not only exhibit intrinsic resistance to antibiotics but also facilitate the persistence of dormant cells and the horizontal transfer of resistance genes. While emerging natural and synthetic antimicrobial agents offer potential avenues for intervention, their effectiveness is often limited by issues such as poor bioavailability, toxicity, and production scalability. To overcome these limitations, the field must shift from incremental refinements to transformative strategies. Promising approaches include electrochemical biofilm disruption, phage-antibiotic synergistic therapies, nanoparticle-mediated delivery systems, CRISPR-based genome editing, natural quorum sensing inhibitors, and the application of next-generation probiotics. However, scientific innovation alone is insufficient. A comprehensive response must also encompass policy reform: implementing strict regulations on antibiotic usage in agriculture, incentivizing the development and adoption of rapid diagnostic tools, and adapting clinical trial designs to support the evaluation of combinatorial and multimodal therapies. Addressing biofilm-associated MDR requires a radical, multidisciplinary approach to effectively counter this growing global threat.

## 1 Introduction

Multidrug resistance (MDR) has transcended the boundaries of a conventional public health issue and now poses a profound existential threat to global healthcare systems and economic stability. With an estimated economic burden projected to reach $100 trillion by 2050 (Haruna et al., 2024), the magnitude of this crisis demands immediate and coordinated international action. Despite these alarming forecasts, current response strategies remain insufficient, often rooted in incremental measures that fail to address the scale and urgency of the problem. Healthcare systems continue to be overwhelmed by MDR pathogens such as methicillin-resistant *Staphylococcus aureus* (MRSA) and carbapenem-resistant Enterobacteriaceae (CRE), which impose significant financial and clinical burdens (Nelson et al., 2021). Simultaneously, unregulated antibiotic use in agriculture continues to accelerate the emergence of resistant strains, undermining both human and animal health. To counter this trajectory, transformative solutions are imperative. This includes substantial government investment in pioneering antimicrobial research, the deployment of AI-enabled surveillance systems in healthcare settings to track resistance patterns in real time, and the advancement of cross-sectoral, multidisciplinary interventions. Critically, MDR must now be recognized as a national and global security threat, given its potential to destabilize food systems, compromise surgical safety, and render modern medical procedures ineffective.

Biofilms, structured microbial communities encased in a self-produced extracellular matrix, are a major contributor to antimicrobial resistance (Safini et al., 2024). These complex aggregates protect bacteria from antibiotics, immune responses, and environmental stresses, making infections chronic and difficult to treat. The rise of MDR pathogens, particularly in hospital-acquired infections, underscores the urgent need for innovative antibiofilm strategies. This article evaluates current trends in combating biofilm-associated MDR, focusing on natural and synthetic compounds, as well as multidisciplinary solutions.

## 2 The role of biofilms in multidrug resistance

**FIGURE 1:**
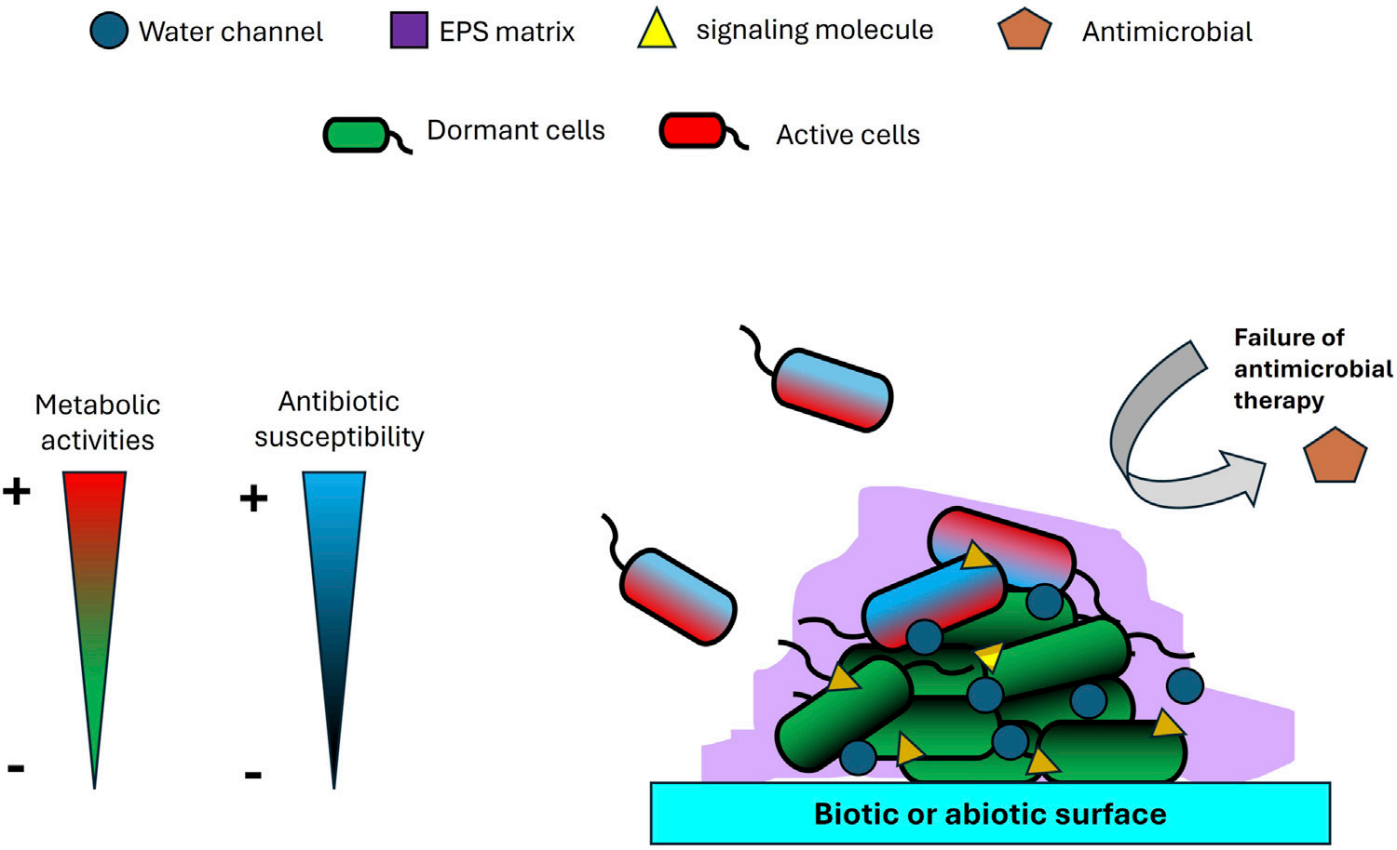
Biofilm structure on biotic or abiotic surface.

Biofilms play a uniquely pivotal role in multidrug resistance (MDR), functioning as highly evolved microbial survival systems that integrate physical, physiological, and genetic defenses into a robust and adaptive architecture (Figure 1). Unlike singular resistance mechanisms such as efflux pumps or enzymatic drug inactivation, biofilms form a complex extracellular matrix composed of polysaccharides, proteins, nucleic acids, and lipids (Yaacob et al., 2021; Kamaruzzaman et al., 2022; Johari et al., 2023; Hamdan et al., 2024; Syaida et al., 2025). This matrix is not merely a passive barrier; it constitutes a dynamic microenvironment capable of modulating external stresses and shielding resident pathogens from antimicrobial agents. One of the most overlooked aspects of biofilm resilience is its metabolic heterogeneity, which facilitates the formation of dormant persister cells, phenotypically tolerant subpopulations that remain unaffected by antibiotics targeting metabolically active cells (Sadiq et al., 2017). Additionally, gradients of oxygen, nutrients, and pH within the biofilm (Sønderholm et al., 2017) create micro-niches that further compromise antimicrobial efficacy. Compounding this issue, the dense structural organization of biofilms accelerates horizontal gene transfer (HGT) (Li et al., 2018), transforming these communities into hotspots for the dissemination of resistance genes. Importantly, biofilm-associated resistance is not solely a function of genetic mutation but rather a complex tolerance phenotype that merges structural fortification with physiological plasticity. This enables pathogens to persist on medical devices and within chronic infections, even when *in vitro* tests suggest susceptibility. Such multifaceted defense mechanisms constitute a form of microbial warfare that challenges conventional treatment paradigms. As such, effective therapeutic strategies must include targeted approaches aimed at disrupting the biofilm matrix, reactivating dormant cells, and inhibiting HGT to counteract this formidable clinical threat.

## 3 Natural compounds: harnessing nature’s arsenal

Natural products represent some of the most promising and underexploited agents in the fight against persistent biofilm-associated infections. Far from being mere alternative therapies, these compounds exhibit intricate multitarget mechanisms that can outmaneuver conventional antibiotics. Phytochemicals such as curcumin, quercetin, and berberine have demonstrated potent quorum sensing (QS) inhibitory activity, disrupting bacterial communication networks and suppressing the biosynthesis of the extracellular matrix (Banerjee et al., 2024). Similarly, essential oils including cinnamaldehyde and eugenol exhibit dual functionality, penetrating biofilm structures while compromising the integrity of multidrug-resistant (MDR) bacterial membranes (Purkait et al., 2020). Particularly noteworthy are enzymatic agents such as Dispersin B and DNase I, which selectively degrade structural components of the biofilm matrix, effectively dismantling its protective barrier (Waryah et al., 2017). Additionally, bacteriophages, nature’s highly evolved antimicrobial agents, have demonstrated the ability to penetrate and lyse bacterial cells within biofilms and exhibit synergistic effects when used in combination with antibiotics (Grygiel et al., 2024). Despite their therapeutic potential, the clinical application of natural products remains limited, largely due to challenges including variability in raw materials, poor bioavailability, and a regulatory environment that heavily favors synthetic compounds. Overcoming these barriers will require a paradigm shift: the adoption of standardized extraction protocols augmented by artificial intelligence, the development of nanocarriers for targeted delivery, and the design of robust clinical trials capable of evaluating combinatorial natural product-based therapies. To effectively address the global burden of biofilm-mediated infections, it is imperative that natural products be repositioned not as adjunctive or alternative options, but as primary, scientifically validated therapeutic tools within our antimicrobial arsenal.

## 4 Synthetic compounds and nanomaterials

Synthetic strategies to combat biofilms offer a high degree of precision and innovation, addressing many of the inherent limitations of conventional antibiotics. Among the most promising approaches are synthetic quorum sensing (QS) inhibitors, such as acyl homoserine lactone (AHL) analogs, which disrupt bacterial communication pathways without inducing the selective pressures that often drive antimicrobial resistance (Majik et al., 2020). These interventions represent a significant paradigm shift in biofilm control, targeting microbial coordination rather than viability. Engineered antimicrobial peptides (AMPs) and their synthetic analogs, peptoids, are emerging as potent anti-biofilm agents due to their ability to penetrate dense biofilm matrices and eliminate persister cells through dual mechanisms involving membrane disruption and intracellular targeting (Stojowska-Swędrzyńska et al., 2023). Equally promising are nanomaterials such as silver, zinc oxide, and graphene-based nanoparticles, which exert multi-faceted antimicrobial activity through the generation of reactive oxygen species (ROS) and direct physical damage to bacterial membranes (Yakup et al., 2024; Yusri et al., 2024). In addition, smart surface coatings on medical devices and implants, designed to resist bacterial adhesion and actively prevent biofilm formation, represent a proactive avenue for reducing device-associated infections (Li et al., 2020). These materials integrate antimicrobial functionality with biocompatibility, offering significant potential in clinical settings. Nonetheless, the widespread application of synthetic strategies must be accompanied by rigorous evaluation of biocompatibility and long-term toxicity, particularly in the context of nanomaterial exposure. Investment in adaptive molecular design and evolution is critical to preempt emerging resistance. Equally important is the development of forward-looking regulatory frameworks that balance the need for innovation with patient safety. If developed and deployed responsibly, synthetic solutions have the potential to revolutionize our approach to biofilm-related infections, providing robust, targeted, and scalable tools in the ongoing battle against antimicrobial resistance.

## 5 Multidisciplinary and AI-driven approaches: the future of antibiofilm therapy

The complex, multicellular nature of biofilms demands integrated therapeutic strategies, as single-mode interventions often fail due to bacterial adaptability and biofilm resilience. Phage-antibiotic synergy (PAS) exemplifies a promising combinatorial approach, wherein bacteriophages lyse biofilm structures and sensitize embedded bacteria (Chaudhry et al., 2017), allowing antibiotics to penetrate and act more effectively, though phage resistance and narrow host specificity remain challenges. Electrochemical disruption, leveraging bioelectric effects to destabilize the extracellular polymeric matrix, offers a non-chemical means to weaken biofilms (Czerwińska-Główka and Krukiewicz, 2020), but its clinical translation requires optimization for tissue-specific applications. Probiotics and microbiome engineering present a biologically nuanced strategy, where commensal bacteria competitively exclude pathogens and secrete secondary metabolites (Torres Salazar et al., 2021), however, strain selection and ecological stability in diverse host environments are critical hurdles. Meanwhile, clustered regularly interspaced palindromic repeats (CRISPR)-based antimicrobials could revolutionize precision therapy by selectively targeting resistance genes or virulence factors in biofilm communities (De la Fuente-Núñez and Lu, 2017), yet delivery mechanisms and off-target effects pose significant barriers to implementation. Collectively, these multimodal approaches highlight the necessity of tailored, context-dependent solutions that address biofilm heterogeneity while mitigating resistance risks.

**FIGURE 2:**
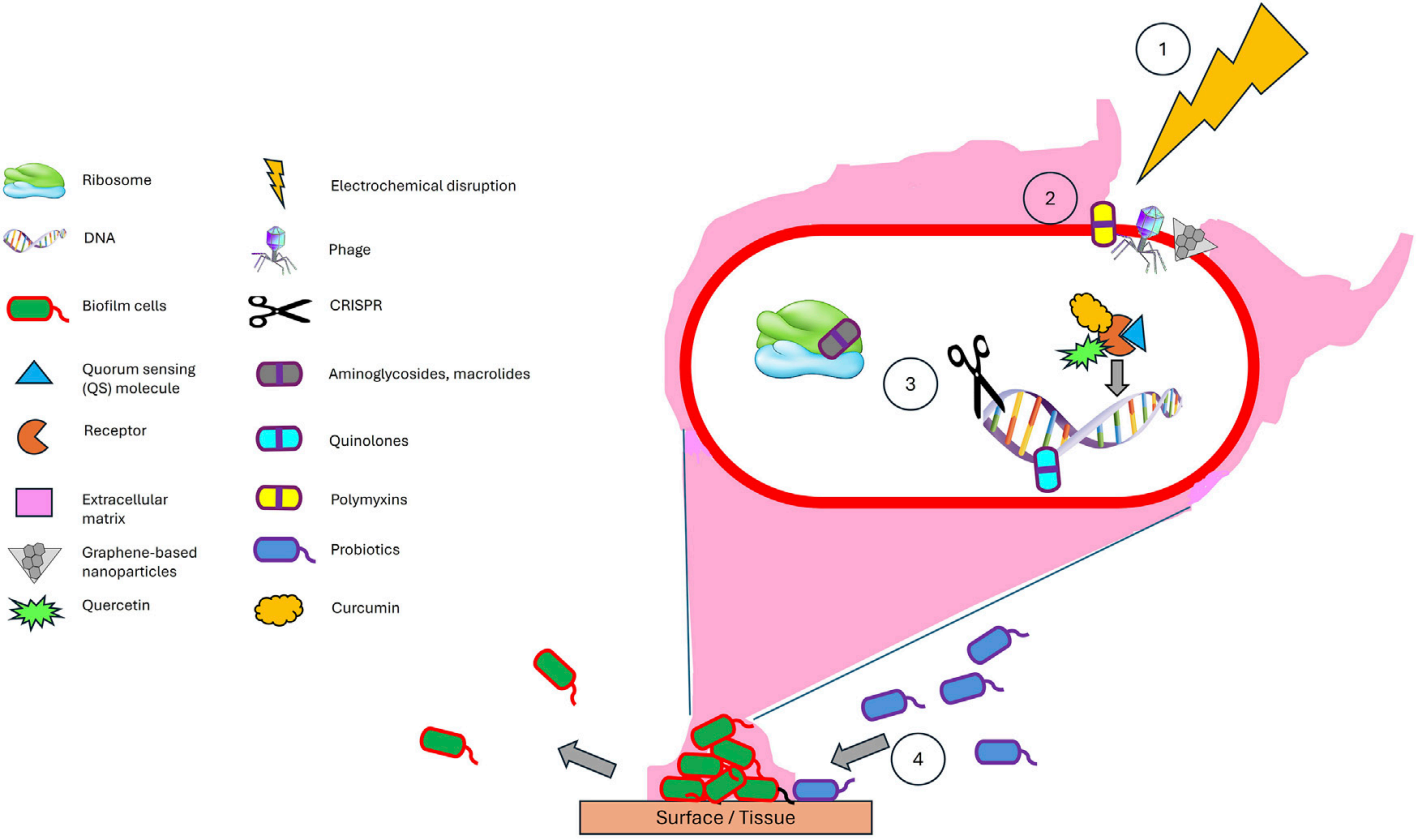
A synergistic combination of antibiofilm strategies - (1) Electrochemical disruption of the matrix facilitates (2) deeper penetration of phages, antibiotics, graphene-based nanoparticles, and natural quorum sensing inhibitors, while (3) CRISPR ensures precise eradication of resistant strains (3), and (4) probiotics provide long-term ecological suppression. This order (1–4) ensures maximal biofilm breakdown, targeted antimicrobial action, and sustainable prevention. This multimodal approach can be effectively adapted for clinical applications, such as treating chronic wounds through electroconductive scaffolds while surgical site infections can be managed via conductive implant coatings to prevent biofilm recurrence. Electrochemical scaffolds and conductive implant coatings offer a programmable, multimodal approach to combat biofilms by first disrupting the biofilm matrix through localized ROS generation, followed by controlled release of antibiotics, quorum sensing inhibitors, phage-nanoparticle conjugates, CRISPR-based tools, and finally integrating probiotics.

A synergistic integration of diverse antibiofilm strategies holds the potential to significantly enhance therapeutic efficacy by leveraging complementary mechanisms of action (Figure 2). For instance, initial electrochemical disruption could compromise the structural integrity of the biofilm matrix, thereby enhancing the penetration and activity of downstream agents such as phage-antibiotic synergy (PAS) systems and graphene-based nanoparticles. These agents can effectively lyse embedded bacteria and sensitize residual populations to both conventional antibiotics and natural quorum sensing inhibitors. Concurrently, CRISPR-based antimicrobials could be employed to selectively target and eliminate resistant or hypervirulent strains, thereby minimizing the risk of regrowth and recurrence. In parallel, the strategic introduction of probiotics may serve to competitively exclude pathogenic species and establish a microenvironment that is antagonistic to biofilm reformation. Metabolites produced by these beneficial microbes could further reinforce the disruption of the biofilm and sustain long-term suppression of pathogenic colonization. This multi-layered, sequential approach enables intervention at various stages of the biofilm lifecycle—structural, genetic, and ecological—offering a comprehensive and adaptable strategy. By exploiting the unique strengths of each modality while offsetting their individual limitations, such a multidisciplinary framework may not only improve clinical outcomes but also reduce the likelihood of resistance development over time.

The proposed combinatorial approach demonstrates strong scientific potential for enhancing antibiofilm efficacy by employing sequential and complementary mechanisms to disrupt, eradicate, and prevent the regrowth of biofilm communities. However, several critical challenges must be addressed to ensure their safe and effective implementation. First, electrochemical parameters require precise calibration to avoid collateral damage to host tissues and resident microbiota. Second, compatibility between bacteriophages and CRISPR-based antimicrobials must be carefully evaluated to prevent unintended off-target gene editing in commensal organisms. Third, the delivery efficiency of phages, antibiotics, natural quorum sensing inhibitors, and graphene-based nanoparticles is contingent upon the degree of biofilm porosity following disruption, an aspect complicated by the inherent structural heterogeneity of biofilms, which can result in uneven drug distribution and residual resistance niches. Finally, the selection of probiotic strains must be strategically guided to prevent antagonistic interactions with phages or antibiotics, thereby maintaining ecological balance and therapeutic synergy. Deploying antibiofilm strategies across diverse environments requires tailored optimization, as factors like temperature, pH, UV exposure, and salinity can significantly influence efficacy. For instance, enzymatic biofilm disruptors may denature at extreme temperatures, while nanoparticle stability can vary with ionic strength in saline conditions (Chopada et al., 2025; Jiang et al., 2025). When optimized, this multidisciplinary strategy offers significant advantages over monotherapies by simultaneously dismantling biofilm architecture, eliminating bacterial populations, suppressing resistance mechanisms, and promoting microbiome stability. Rigorous preclinical evaluation is essential to validate the efficacy and safety of this approach across diverse infection models. Notably, Morrisette et al. (2020) suggest that such combination therapies may enable reduced antibiotic dosages, thereby minimizing selective pressure for resistance. Similarly, prior studies advocate multi-targeted or combinatorial regimens as more effective strategies for overcoming the resilience of biofilm-associated infections (Koo et al., 2017; Ranall et al., 2012). Emerging technologies such as stimuli-responsive drug delivery systems, metal-organic frameworks, and hydrogel-based carriers further enhance the prospects of this integrated approach by improving localized delivery, bioavailability, and controlled release (Sikder et al., 2021). Taken together, these innovative strategies mark a pivotal evolution in antimicrobial design and deployment, offering a compelling path forward in addressing the escalating global crisis of antibiotic-resistant infections.

Artificial intelligence (AI) is revolutionizing the development and optimization of multimodal antibiofilm strategies by enabling data-driven decision-making at multiple levels. According to Fagbemi et al. (2025), AI tools can significantly enhance the development of antibiofilm strategies by analyzing vast datasets to predict effective drug combinations, optimize treatment sequences, and identify novel biofilm-disrupting targets. AI-powered systems can optimize antibiotic use by recommending effective treatments based on patient data and local resistance patterns, while also accelerating drug discovery (Branda and Scarpa, 2024). AI-driven approaches such as deep learning can analyze genomic, proteomic, and metabolomic data to pinpoint key biofilm-related genes or vulnerable metabolic pathways for targeted interventions (Yetgin, 2025). Additionally, AI-driven approaches enable rapid screening of parameters to discover optimal nanoparticles configurations for specific therapeutic needs (Kapoor et al., 2024). However, challenges include the need for high-quality, standardized datasets, model interpretability, and real-world validation to ensure clinical applicability. Integrating AI with experimental and clinical data will be crucial for accelerating the translation of predictive insights into effective antibiofilm therapies.

## 6 Conclusion

To effectively confront biofilm-mediated multidrug resistance (MDR), it is imperative that we move beyond our continued reliance on conventional antibiotics. A paradigm shift in therapeutic strategy is urgently required. While natural compounds offer considerable promise due to their ecological safety and broad-spectrum mechanisms of action, the precision and tunability of synthetic and nanotechnology-based antimicrobials represent a transformative advancement. The future of antibiofilm therapy will likely depend on the strategic integration of these modalities with advanced delivery platforms to enhance efficacy and overcome physiological barriers. However, significant challenges remain. Regulatory complexities, high development costs, and limited scalability continue to impede the clinical translation of these innovations. Despite robust scientific progress, the deployment of next-generation antibiofilm therapies remains constrained by systemic inertia. To bridge the gap between laboratory breakthroughs and real-world applications, we must align scientific innovation with streamlined regulatory frameworks, sustainable funding mechanisms, and policy-level commitment.

## Data Availability

The original contributions presented in the study are included in the article/supplementary material, further inquiries can be directed to the corresponding author.
